# Inhibitory effect of *N*-ethyl-3-amino-5-oxo-4-phenyl-2,5-dihydro-1*H*-pyrazole-1-carbothioamide on *Haemophilus* spp. planktonic or biofilm-forming cells

**DOI:** 10.1007/s00044-013-0700-7

**Published:** 2013-08-23

**Authors:** Urszula Kosikowska, Anna Malm, Monika Pitucha, Barbara Rajtar, Malgorzata Polz-Dacewicz

**Affiliations:** 1 Department of Pharmaceutical Microbiology, Medical University of Lublin, Chodzki Str. 1, 20-093 Lublin, Poland; 2 Department of Organic Chemistry, Medical University, Chodzki Str. 4a, 20-093 Lublin, Poland; 3 Department of Virology, Medical University, Chodzki Str. 1, 20-093 Lublin, Poland

**Keywords:** *Haemophilus* spp., Pyrazole derivatives, Antibacterial activity, Anti-biofilm activity, Cytotoxicity

## Abstract

During this study, we have investigated in vitro activity of *N*-substituted-3-amino-5-oxo-4-phenyl-2,5-dihydro-1*H*-pyrazole-1-carbothioamide derivatives with *N*-ethyl, *N*-(4-metoxyphenyl) and *N*-cyclohexyl substituents against Gram-negative *Haemophilus influenzae* and *H*. *parainfluenzae* bacteria. A spectrophotometric assay was used in order to determine the bacterial growth and biofilm formation using a microtiter plate to estimate minimal inhibitory concentration (MIC) and minimal biofilm inhibitory concentration (MBIC). Among the tested *N*-substituted pyrazole derivatives, only *N*-ethyl-3-amino-5-oxo-4-phenyl-2,5-dihydro-1*H*-pyrazole-1-carbothioamide showed a significant in vitro activity against both planktonic cells of *H*. *parainfluenzae* (MIC = 0.49–31.25 μg ml^−1^) and *H*. *influenzae* (MIC = 0.24–31.25 μg ml^−1^) as well as biofilm-forming cells of *H*. *parainfluenzae* (MBIC = 0.24–31.25 μg ml^−1^) and *H. influenzae* (MBIC = 0.49 to ≥31.25 μg ml^−1^). The pyrazole compound exerted higher inhibitory effect both on the growth of planktonic cells and biofilm formation by penicillinase-positive and penicillinase-negative isolates of *H. parainfluenzae* than the activity of commonly used antibiotics such as ampicillin. No cytotoxicity of the tested compound in vitro at concentrations used was found. The tested pyrazole *N*-ethyl derivative could be considered as a compound for the design of agents active against both pathogenic *H. influenzae* and opportunistic *H. parainfluenzae*, showing also anti-biofilm activity. This appears important because biofilms are determinants of bacterial persistence in long-term and recurrent infections recalcitrant to standard therapy.

## Introduction

Biofilms are sessile aggregates of bacterial cells that are created on either biotic surfaces (e.g., human tissues) or abiotic surfaces (e.g., biomaterials, catheters) and act like a single living organism that can exhibit differences in the expression of surface molecules, antimicrobial resistance, virulence factors, and pathogenicity (Costerton *et al.*, 1999, 2003; Burmølle *et al.*, 2010; Hall-Stoodley *et al.*, 2012; Bjarnsholt, 2013). In medicine, biofilms have been widely associated with several chronic and recurrent diseases, chronic wound infections, and foreign body infections associated with implantable medical devices and indwelling catheters, antibiotic-resistant and nearly impossible or difficult to eradicate without aggressive and long-term interventional strategies infections (Donlan, 2001; Steward and Costeron, 2001; Gilbert *et al.*, 2002; Stoodley *et al.*, 2004; Lasa *et al.*, 2005; Sanclement *et al.*, 2005; Macfarlane and Dillon, 2007; Vlastarakos *et al.*, 2007; Macedo and Abraham, 2009; Wolcott and Ehrlich, 2008; Coenye and Nelis, 2010; Drago *et al.*, 2012; Bjarnsholt, 2013). 

*Haemophilus* spp. rods, generally known as Gram-negative microbiota of the upper respiratory tract, are able to live as planktonic cells or colonize natural and artificial surfaces as biofilm-forming cells (Hill *et al.*, 2000; Chin *et al.*, 2005; Musk and Hergenrother, 2006; Galli *et al.*, 2007; Kilian, 2007; Moxon *et al.*, 2008; Kosikowska and Malm, 2009; Murphy *et al.*, 2007; Drago *et al.*, 2012; Ünal *et al.*, 2012). Both pathogenic *Haemophilus influenzae* and opportunistic *H. parainfluenzae* can cause acute, chronic, invasive or non-invasive infections. These microorganisms may form a biofilm which is a virulence determinant which contributes to recurrent or chronic infections. *H.* *influenzae* is the most pathogenic bacteria colonizing the mucous membranes of the respiratory tract of young children or sporadically elderly people. *H. influenzae*, mainly serotype b (Hib), is frequently associated with different diseases, e.g., otitis media, chronic bronchitis, and pneumonia (Agrawal and Murphy, 2011). Other illnesses caused by this species include osteomyelitis, arthritis, sepsis, phlegmon cellulitis, or abscesses. Non-typeable *H. influenzae* (NTHi) is one of the main causes of airway infection in chronic obstructive pulmonary disease, of recurrent otitis media in infants and children, sinusitis in children and adults, pneumonia in adults, lower respiratory tract infection in adults, and recurrent respiratory tract infections in patients with chronic bronchitis (Murphy, 2003; Erwin and Smith, 2007).

*Haemophilus parainfluenzae* is an opportunistic pathogen, which may cause several endogenous diseases occasionally and under predisposing conditions (e.g., chronic diseases or immune disorders) such as respiratory tract infections, endocarditis, biliary tract infection, septic arthritis, thoracic empyema, meningitis, secondary bacteremia, urethritis, and hepatic abscesses (Chow *et al.*, 1974; Cooney *et al.*, 1981; Warman *et al.*; 1981; Raoult *et al.*, 1987; Darras-Joly *et al.*, 1997; Das *et al.*, 1997; Bottone and Zhang,^1995^; Pillai *et al.*, 2000; Frankard *et al.*, 2004; Cardines *et al.*, 2009).

Nitrogen heterocycles, including pyrazoles, are important group of natural or synthetic derivatives with a broad spectrum of biological and pharmaceutical activities, e.g., antibacterial, antifungal, antiviral, anti-inflammatory, antipyretic, anticancer, and anticonvulsant (Comber *et al.*, 1991; Mahajan *et al.*, 1991; Chauhan *et al.*, 1993; Sugiura et al. 1977; Bekhit and Abdel-Aziem, 2004; Gökhan-Kelekçi *et al.*, 2007; Lin *et al.*, 2007; Kumar *et al.*, 2012). Much attention has been paid to pyrazole derivatives due to their wide range of antibacterial activities as potential and selective inhibitors against DNA gyrase capable of causing bacterial cells’ death (Reece and Maxwell, 1991; Maxwell, 1997; Tanitame *et al.*, 2004; Liu *et al.*, 2008; Shiroya *et al.*, 2011). *N*-ethyl-3-amino-5-oxo-4-phenyl-2,5-dihydro-1*H*-pyrazole-1-carbothioamide, synthesized according to Pitucha *et al.* (2010) appears to be a promising precursor of agents with good activity mainly against Gram-positive bacteria––both pathogenic, including *Staphylococcus* *aureus* (MIC = 7.81–62.5 μg ml^−1^) as well as opportunistic, e.g., *S. epidermidis*, *Bacillus* spp. or *Micrococcus luteus* with MIC = 3.91–31.25 μg ml^−1^ (Pitucha *et al.*, 2010). The inhibitory effect against Gram-negative bacteria––belonging to *Enterobacteriaceae* family (*Escherichia coli*, *Klebsiella pneumoniae*, and *Proteus mirabilis*) or nonfermentative rods (*Pseudomonas aeruginosa*) was weaker (MIC = 250–1,000 μg ml^−1^). In this paper, we have investigated in vitro activity of pyrazole derivatives, among which *N*-ethyl-3-amino-5-oxo-4-phenyl-2,5-dihydro-1*H*-pyrazole-1-carbothioamide showed highest activity against planktonic and biofilm-forming cells of *H. influenzae* and *H. parainfluenzae.*

## Results and discussion

According to our preliminary results, *N*-(4-metoxyphenyl)-3-amino-5-oxo-4-phenyl-2,5-dihydro-1*H*-pyrazole-1-carbothioamide compound had very low inhibitory effect on the growth of planktonic cells of the reference strains of *Haemophilus* spp. (MIC = 500–1,000 μg ml^−1^), similar to *N*-cyclohexyl-3-amino-5-oxo-4-phenyl-2,5-dihydro-1*H*-pyrazole-1-carbothioamide which inhibited the growth of these bacteria with somewhat lower MIC = 125–500 μg ml^−1^. Among the tested pyrazole derivatives, *N*-ethyl-3-amino-5-oxo-4-phenyl-2,5-dihydro-1*H*-pyrazole-1-carbothioamide derivative showed a significant in vitro potency against the growth of planktonic cells of the tested *Haemophilus* spp. strains with MIC <62.5 μg ml^−1^.

As shown in Table 1, detailed studies with *N*-ethyl-3-amino-5-oxo-4-phenyl-2,5-dihydro-1*H*-pyrazole-1-carbothioamide revealed that this compound possessed good activity against planktonic cells of the reference strains of *H. parainfluenzae* ATCC 7901 (MIC = 0.49 μg ml^−1^), *H. parainfluenzae* ATCC 51505 (MIC = 7.81 μg ml^−1^), and *H. influenzae* ATCC 10211 (MIC = 0.49 μg ml^−1^). This compound was also active against planktonic cells of 20 clinical isolates of *H. parainfluenzae* (MIC = 1.95–31.25 μg ml^−1^) and of 11 clinical isolates of *H. influenzae* (MIC = 0.24–31.25 μg ml^−1^). Moreover, the activity of the tested compound against *H. parainfluenzae* and *H. influenzae* biofilm-forming cells was also determined––it inhibited biofilm formation by reference strains of *H. parainfluenzae* ATCC 7901 (minimal biofilm inhibitory concentration [MBIC] = 1.95 μg ml^−1^) and *H. parainfluenzae* ATCC 51505 (MBIC = 15.63 μg ml^−1^) or by 20 clinical isolates of *H. parainfluenzae* (MBIC = 0.24–31.25 μg ml^−1^). The tested compound showed the inhibitory effect against biofilm-forming cells of *H. influenzae* ATCC 10211 (MBIC = 15.63 μg ml^−1^) or seven *H. influenzae* clinical isolates (MBIC = 0.49–31.25 μg ml^−1^). In case of four clinical isolates of *H. influenzae*, MBIC were found to be >31.25 μg ml^−1^.

**Table 1:** The effect of *N*-ethyl-3-amino-5-oxo-4-phenyl-2,5-dihydro-1*H*-pyrazole-1-carbothioamide on the growth of *Haemophilus* spp. planktonic (MIC) or biofilm-forming (MBIC) cells

Species		Growth		Biofilm formation	
		MIC (μg ml^−1^)	No. of strains	MBIC (μg ml^−1^)	No. of strains
*Haemophilus parainfluenzae*	ATCC 7901	0.49	1	1.95	1
	ATCC 51505	7.81	1	15.63	1
	Clinical isolates (n = 20)	0.24	0	0.24	1
		0.98	0	0.98	1
		1.95	1	1.95	3
		3.91	1	3.91	3
		7.81	3	7.81	0
		15.63	7	15.63	6
		31.25	8	31.25	6
*Haemophilus influenzae*	ATCC 10211	0.49	1	15.63	1
	Clinical isolates (n = 11)	0.24	1	0.24	0
		0.49	1	0.49	1
		0.98	3	0.98	1
		1.95	1	1.95	2
		3.91	1	3.91	1
		7.81	0	7.81	1
		15.63	2	15.63	0
		31.25	2	31.25	1
		>31.25	0	>31.25	4

**Fig. 1:**
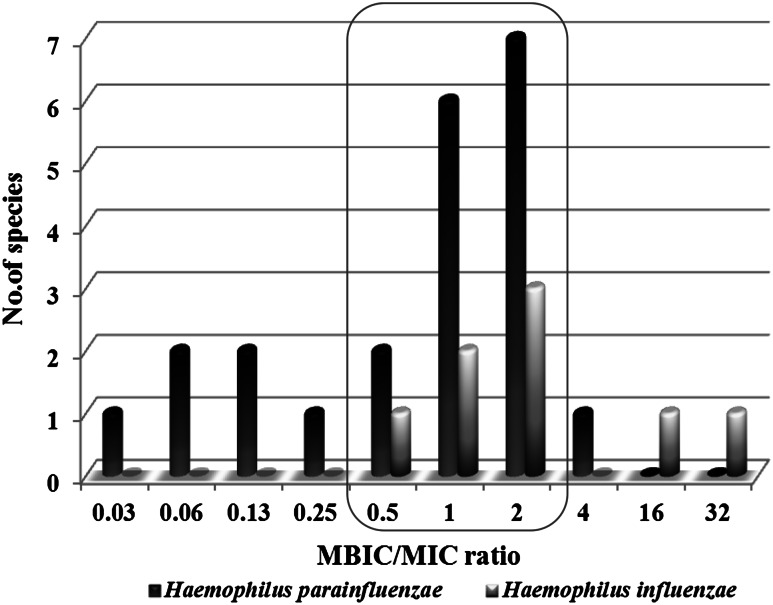
The effect of *N*-ethyl-3-amino-5-oxo-4-phenyl-2,5-dihydro-1*H*-pyrazole-1-carbothioamide compound on biofilms formation by *Haemophilus* spp. on the basis of MBIC/MIC ratio

To determine the power of the tested compound as an anti-biofilm agent, the MBIC/MIC ratio was assessed. The most frequently MBIC/MIC ratio ranged from 0.5 to 2 μg ml^−1^, indicating comparable activity of the compound either against planktonic or biofilm-forming cells of *H. parainfluenzae* and *H. influenzae* (Fig. 1). Only in some cases, MBIC/MIC ratio was lower for *H. parainfluenzae* and was higher for *H. influenzae*, indicating strain- and species-dependent activity of the tested compound.

**Fig. 2:**
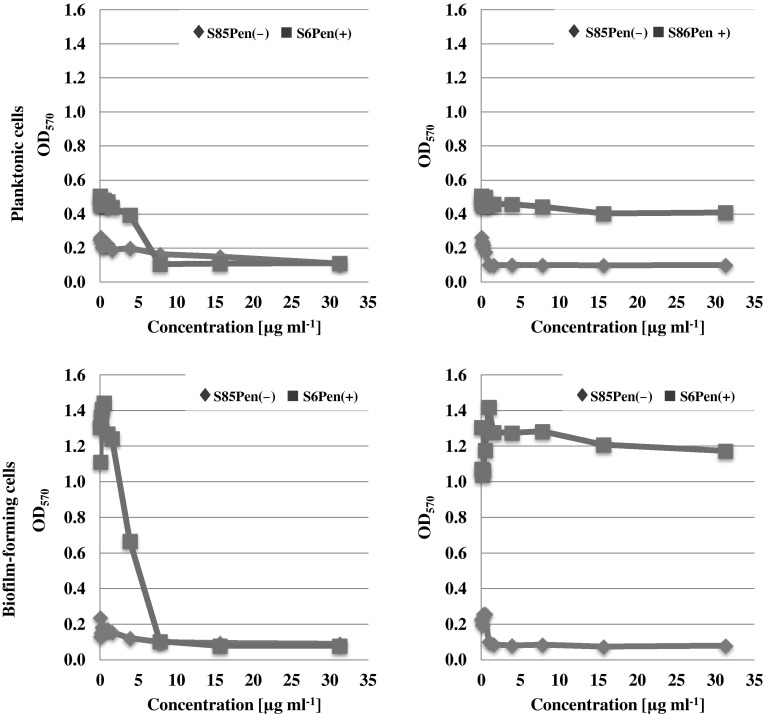
The effect of *N*-ethyl-3-amino-5-oxo-4-phenyl-2,5-dihydro-1*H*-pyrazole-1-carbothioamide compound and ampicillin on the penicillinase-negative (*filled diamond* S85Pen−) and penicillinase-positive (*filled square* S86Pen+) *Haemophilus parainfluenzae* planktonic or biofilm-forming cells (broth without bacteria: OD_570_ = 0.09–0.11)

Figure 2 shows the activity of *N*-ethyl-3-amino-5-oxo-4-phenyl-2,5-dihydro-1*H*-pyrazole-1-carbothioamide on the growth or biofilm formation by penicillinase-negative (S85Pen−) and penicillinase-positive (S86Pen+) *H. parainfluenzae*. In the case of penicillinase-positive isolate, the activity of the compound was significantly higher both on the growth and on the biofilm formation.

The in vitro cytotoxicity of the tested *N*-ethyl-3-amino-5-oxo-4-phenyl-2,5-dihydro-1*H*-pyrazole-1-carbothioamide compound was presented as percentage viability of vero cells used as an experimental model. According to the results shown in Table 2, after 48 h of incubation, no cytotoxic effect was observed up to 200 μg ml^−1^ concentration of the tested compound. The most widely used as a measurement of compound’s toxicity is the half maximal effective concentration (EC_50_), as the concentration of the compound where 50 % of its maximal effect is observed; in case of the tested compound EC_50_ = 278.8 μg ml^−1^. This means that this compound was not toxic to eukaryotic cells at concentrations exerting inhibitory effect against *Haemophilus* spp., including anti-biofilm activity.

**Table 2:** The effect of *N*-ethyl-3-amino-5-oxo-4-phenyl-2,5-dihydro-1*H*-pyrazole-1-carbothioamide on vero cells line viability

Compound concentration (μg ml^−1^)	Cell viability (in %) ± SD
	*x*
500	37.95 ± 7.7
200	82.15 ± 5.7
100	89 ± 6.6
50	93.55 ± 4.2
25	97.4 ± 1.7
12.5	98.05 ± 1.8
6.25	98.75 ± 2.0
3.15	100 ± 0.0
0 (control)	100 ± 0.0

Although the control of bacterial infections has been effective since the discovery of antimicrobial drugs, widespread drug resistance among bacteria has led to a search for new antibacterial agents. However, the finding of biofilm phenotype bacteria, showing usually intrinsic insensitivity to available drugs at standard dosing effective against planktonic cells, has created a necessity to pay more attention to targeted anti-biofilm agents. In this work, we have found that the *N*-ethyl-3-amino-5-oxo-4-phenyl-2,5-dihydro-1*H*-pyrazole-1-carbothioamide possessed good in vitro activity either against free-floating (planktonic) or biofilm-forming cells of *Haemophilus* spp. Haemophili rods, e.g., pathogenic *H. influenzae* or opportunistic *H. parainfluenzae* are found to be a part of proper microflora of the upper respiratory tract (Kilian, 2007; Murphy *et al.*, 2007). Under favorable conditions, these bacterial species may be the etiologic agents of various and unspecified infections, including those associated with biofilm formation (Trollfors *et al.*, 1985; Black *et al.*, 1988; Mitchell and Hill, 2000; Chin *et al.*, 2005; Musk and Hergenrother, 2006; Rele *et al.*, 2006; Galli *et al.*, 2007; Moxon *et al.*, 2008; Cardines *et al.*, 2009; Drago et al., 2012; Bjarnsholt, 2013). It has been estimated that the biofilms protect microbes from the immune system, antimicrobials, predation or stresses, and are crucial for the development of recurrent and opportunistic diseases (Costerton *et al.*, 1999, 2003; Donlan, 2002; Prakash *et al.*, 2003; Jain *et al.*, 2007; Wolcott and Ehrlich, 2008).

The pyrazole derivatives are potent and selective inhibitors against DNA gyrase (Reece and Maxwell, 1991; Tanitame *et al.*, 2004; Tse-Dinh, 2007; Farag *et al.* 2008; Liu *et al.*, 2008; Shiroya *et al.*, 2011). Considering a possible mechanism of anti-biofilm activity of *N*-ethyl-3-amino-5-oxo-4-phenyl-2,5-dihydro-1*H*-pyrazole-1-carbothioamide, it should be noted that several classes of chemical compounds, e.g., pyrazole or thioamide derivatives, may act as quorum-sensing inhibitors (Hentzer and Givskov, 2003; Schillaci *et al.* 2008; Brackman *et al.*, 2009; Kociolek, 2009; Oancea, 2010). Quorum-sensing phenomenon, which is one of the ways to control biofilms, is a chemical form of bacterial communication via signaling molecules essential for bacterial communities to regulate the group and to synchronize the behavior (Hastings and Greenberg, 1999; Van Houdt *et al.*, 2004; Raffa *et al.*, 2005; Waters and Bassler, 2005; Musk and Hergenrother, 2006; Bjarnsholt and Givskov, 2007; Amer *et al.*, 2008; Labandeira-Rey *et al.*, 2009; Deep *et al.*, 2011). In agreement with the data provided by the literature, pyrazole compounds may act as inhibitors that target this cell–cell signaling mechanism (Tanitame *et al.*, 2004; Musk and Hergenrother, 2006; Tse-Dinh, 2007; Schillaci *et al.*, 2008; Brackman *et al.*, 2009; Oancea, 2010). The number of literature data dealing with regulatory mechanisms controlling the haemophili biofilm formation and a possible effect of different chemical compounds on this process is strongly limited. In our opinion, comparable activity of the tested compound having the ethyl substituent against planktonic or biofilm-forming cells of haemophili rods may be due to the dual activity of pyrazole––main inhibitory effect against DNA gyrase and additional activity associated with the disorder of quorum-sensing phenomenon and biofilm formation. We did not find existing studies dealing with effect of the pyrazole compounds on formation or eradication of biofilms created by *H. influenzae* and *H. parainfluenzae*.

It should be mentioned that Lux-S family of quorum-sensing regulatory systems involved in production of autoinducer 2 (AI-2), occurring in many bacterial species and functioning as interspecies signaling system, have been identified in *H. influenzae* or *H. ducrei* (Bassler, 1999; Vendeville *et al.*, 2005; Armbruster *et al.*, 2009; Swords, 2012). Ünal *et al.* (2012) have showed that in nontypeable *H. influenzae*, the two-component signaling system QseB/C was involved in biofilm formation. Daines *et al.* (2005) and Armbruster *et al.* (2009) have observed the role of LuxS and AI-2 *luxS*-dependent factors which control biofilm formation in non-typable *H. influenzae*, but they considered as controversial its importance as virulence factor in pathogenesis of the biofilm-associated infections.

The change in the structure of the substituent has a significant impact on the physicochemical properties of the compound (Hulzebos *et al.*, 2001; Martin *et al.*, 2002). In our study, we synthesized and tested derivatives differing from each other by the type of substituents in the thioamide group. The best results were obtained for the compound having the ethyl substituent. From the microbiological point of view, the key factor is the presence of ethyl group which only slightly increases the mass and the volume of the compound compared to derivatives with cyclohexyl or 4-metoxyphenyl substituents. Additionally, lower molecular weight of ethyl derivative can have a significant effect on the antimicrobial properties of this compound. In our opinion, replacement of ethyl group on cyclohexyl or 4-metoxyphenyl in the tested pyrazole derivatives causes a significant decrease of their activity against *Haemophilus* spp. Besides, ethyl substituent has a limited conformational freedom which may affect selectivity (Graham, 2001). This is very important information from the point of view of the further modifications of these derivatives and their activity against either biofilm-forming cells or against mature biofilm of *Haemophilus* spp. In addition, further work is needed to evaluate the role of pyrazole derivatives during biofilm formation and their influence either on adhesive capabilities of haemophili rods or on quorum-sensing phenomenon.

## Conclusions

*N*-ethyl-3-amino-5-oxo-4-phenyl-2,5-dihydro-1*H*-pyrazole-1-carbothioamide, *N*-(4-metoxyphenyl)-3-amino-5-oxo-4-phenyl-2,5-dihydro-1*H*-pyrazole-1-carbothioamide, and *N*-cyclohexyl-3-amino-5-oxo-4-phenyl-2,5-dihydro-1*H*-pyrazole-1-carbothioamide were tested against *H. influenzae* and *H. parainfluenzae* in form of planktonic or biofilm-forming cells. Our study shows that the pyrazoles can be inhibitors acting on planktonic or biofilm-forming cells of *Haemophilus* spp. Additionally, these results allow to expect that this compound will be the starting substance in the search of antimicrobials with low toxicity, showing inhibitory effect against Gram-negative haemophili rods and including anti-biofilm activity. Further investigations should clarify the mechanism of pyrazoles against biofilm formed by haemophili rods.

## Materials and methods

### *N*-substituted-3-amino-5-oxo-4-phenyl-2,5-dihydro-1*H*-pyrazole-1-carbothioamide derivatives

Three *N*-substituted-3-amino-5-oxo-4-phenyl-2,5-dihydro-1*H*-pyrazole-1-carbothioamide derivatives have been screened for the antibacterial investigations. Obtained compounds were characterized and described earlier (Pitucha *et al.*, 2010).

**Scheme 1:**
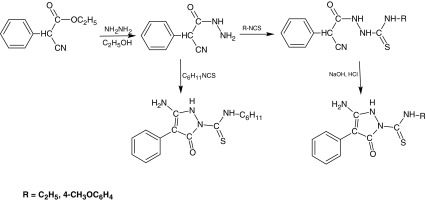
Synthesis and structure of *N*-substituted-3-amino-5-oxo-4-phenyl-2,5-dihydro-1*H*-pyrazole-1-carbothioamide

*N*-substituted-3-amino-5-oxo-4-phenyl-2,5-dihydro-1*H*-pyrazole-1-carbothioamide derivatives were prepared according to Scheme 1. The starting 1-cyanophenylacetic acid hydrazide was prepared in the reaction of corresponding ethyl 1-cyanophenylacetate with 80 % hydrazine hydrate at room temperature. Next, this compound was converted to the 1-(cyanophenylacetyl-4-subtituted)thiosemicarbazide in the reaction of 1-cyanophenylacetic acid hydrazide with ethyl or 4-methoxyphenyl isothiocyanate. Cyclization of these compounds in alkaline or hydrochloric acid medium led to appropriate *N*-substituted-3-amino-5-oxo-4-phenyl-2,5-dihydro-1*H*-pyrazole-1-carbothioamide. *N*-cyclohexyl-3-amino-5-oxo-4-phenyl-2,5-dihydro-1*H*-pyrazole-1-carbothioamide was obtained in the reaction of 1-cyanophenylacetic acid hydrazide with cyclohexyl isothiocyanate. The reaction was carried out in the diethyl ether at room temperature without the separation of linear product.

### Bacterial strains

The haemophili reference species from American Type Culture Collection (ATCC)––*H. influenzae* ATCC 10211, *H. parainfluenzae* ATCC 7901, and *H.* *parainfluenzae* ATCC 51505 were included. Besides, 20 clinical isolates of *H. parainfluenzae* and 11 clinical isolates of *H. influenzae* from the museum of Department of Pharmaceutical Microbiology of Medical University of Lublin were used.

### Growth conditions

The *Haemophilus* chocolate agar (HAEM, bioMerieux) medium with PolyVitex and hemoglobin or tripticasein soy broth (TSB) + *Haemophilus* test medium supplement (HTMS)––TSB (Biocorp) medium supplemented with HTMS (HTMS SRO158E, Oxoid) with growth factors for haemophili (25 μg ml^−1^ of NAD and 15 μg ml^−1^ of hematin) were used. Chocolate agar is blood agar medium that has been heated to open the pyrrole ring, forming haemin (a required growth factor for bacteria lacking hemolysins), providing optimal growth conditions for *H. influenzae* and other fastidious bacteria (Rennie *et al.*, 1992; Han *et al.*, 2006). In clinical microbiology, the TSB medium is used in a variety of procedures, e.g., for the microbiological test procedure of culture media according to the standards (NCLSI, 2000, 2004). However, according to our results, TSB supplemented with HTMS is good as a primary enrichment medium directly inoculated with the various bacteria (Kosikowska and Malm, 2009). The standardized bacterial suspensions with an optical density of 0.5 McFarland standard––150 × 10^6^ colony-forming units ml^−1^ in sterile 0.85 % NaCl were prepared. A stock solutions of *N*-substituted-3-amino-5-oxo-4-phenyl-2,5-dihydro-1*H*-pyrazole-1-carbothioamide derivatives at a concentration of 50 mg ml^−1^ in dimethyl sulfoxide (Sigma) were prepared. The medium with DMSO at a final concentration and without the tested compounds served as control––no microbial growth inhibition was observed.

The bacterial cultures in TSB+HTMS medium with addition of 30 % glycerol were stored at −70 °C. Before each experiment, bacterial strains were subcultured on HAEM medium and incubated overnight at 35 °C in about 5 % CO_2_ atmosphere.

### Growth assay

Preliminary in vitro antibacterial activity of compounds *N*-ethyl-3-amino-5-oxo-4-phenyl-2,5-dihydro-1*H*-pyrazole-1-carbothioamide, *N*-(4-metoxyphenyl)-3-amino-5-oxo-4-phenyl-2,5-dihydro-1*H*-pyrazole-1-carbothioamide, and *N*-cyclohexyl-3-amino-5-oxo-4-phenyl-2,5-dihydro-1*H*-pyrazole-1-carbothioamide was screened by the broth microdilution method using 96-well polystyrene microplates (NUNC, TC MICROWELL 96F Nunclon D) on the basis of MIC (minimal inhibitory concentration), usually defined as the lowest concentration of the compounds at which there was no visible growth of microorganisms. The antibacterial activity was tested according to EUCAST (2003) procedure with some modifications.

In order to assay the influence of the tested pyrazole derivatives on the growth of haemophili rods, 198 μl of TSB+HTMS medium without (control) and with a series of twofold dilution of the tested compounds in the range of final concentration from 1,000 to 62.5 μg ml^−1^ was inoculated with 2 μl of the standardized microbial suspension (total volume per each well ––200 μl), and then incubated for 18 h at 35 °C in the presence of about 5 % CO_2_. Then in order to assay the influence of the tested *N*-ethyl-3-amino-5-oxo-4-phenyl-2,5-dihydro-1*H*-pyrazole-1-carbothioamide with highest inhibitory effect against the planktonic cells of *Haemophilus* spp., 198 μl of TSB+HTMS medium without (control) and with a series of twofold dilution of the tested compound in the range of final concentration from 0.12 to 31.25 μg ml^−1^ was inoculated with 2 μl of the standardized microbial suspension (total volume per each well––200 μl), and then incubated for 18 h at 35 °C in the presence of about 5 % CO_2_. After incubation, spectrophotometric measurements of optical density at wavelength *λ* = 570 (OD_570_) of the bacterial cultures with or without the tested compound were done by using a microplate reader (ELx800 BioTek) in order to determine MIC. The MIC values were determined by comparison to the growth of a control (compound-free) medium. Ampicillin was used as a reference antimicrobial agent on selected *H. parainfluenzae* (penicillinase-positive or penicillinase-negative strains) isolates at the same conditions. The blank control wells without or with twofold dilution of the tested compounds added to TSB+HTMS broth without bacterial suspension were incubated under the same conditions. The experiments were performed in triplicate.

### Biofilm assay

In order to assay the effect of *N*-ethyl-3-amino-5-oxo-4-phenyl-2,5-dihydro-1*H*-pyrazole-1-carbothioamide on *Haemophilus* spp. biofilm formation, the method based on staining with 0.1 % crystal violet described previously by Kaplan and Mulks (2005) with some modifications (Kosikowska and Malm, 2009) was used. The activity of the tested compound against biofilm formation was determined on the basis of MBIC, defined as the lowest concentration of the compound at which the biofilm formation was inhibited (Černohorská and Votava, 2008). In order to assay the influence of the tested compound on the biofilm formation by haemophili rods, 198 μl of TSB+HTMS medium without (control) and with a series of twofold dilution of the tested compound in the range of final concentration from 0.12 to 31.25 μg ml^−1^ was inoculated with 2 μl of the standardized microbial suspension (total volume per each well––200 μl), and then incubated at 35 °C in the presence of about 5 % CO_2_. After overnight incubation of bacterial culture, the medium above the culture was decanted and then the plates were washed extensively several times with distilled water to remove nonadherent or loosely adherent cells, dried in inverted position and stained with 200 μl of 0.1 % crystal violet. The plates were left for 15 min to stain the cells, then washed extensively under distilled water to remove unbound dye. Next, in order to elicit a response to each of the wells, 200 μl of isopropyl alcohol (Color Gram 2 R 3-F, bioMerieux) was added and the plates were left at room temperature for 15 min to solubilize the dye. The optical density of the alcohol–dye solution in each well was read at wave length *λ* = 570 (OD_570_) by using a microplate reader (BioTek ELx800). Ampicillin was used as a reference compound. The blank control wells without or with twofold dilution of the tested compound added to TSB+HTMS broth without bacterial suspension were incubated under the same conditions. The experiments were performed in triplicate.

### Cytotoxicity assay

The vero cell culture from the American Type Culture Collection (ATCC––84113001) was used in the experiment. The minimum essential medium Eagle (MEM, Sigma) media were supplemented with 10 % fetal bovine serum (Sigma), 100 U ml^−1^ of penicillin, and 0.1 μg ml^−1^ of streptomycin (Polfa-Tarchomin, Poland). The cell culture was incubated at 37 °C for 24 h in the 5 % CO_2_ atmosphere. A stock solution of *N*-ethyl-3-amino-5-oxo-4-phenyl-2,5-dihydro-1*H*-pyrazole-1-carbothioamide at a concentration of 50 mg ml^−1^ was dissolved in DMSO (Sigma). The initial concentration of the examined compound in the MEM medium was 500 μg ml^−1^.

100 μl of the vero cell culture prepared was plated into 96-well polystyrene microplates (NUNC) at a cell density 2 × 10^4^ cells per well. After 24 h incubation at 37 °C, the media were removed and the cells were treated with a solution of the tested compound diluted in the MEM medium including 2 % of serum. The following final concentrations were applied: 3.15, 6.25, 12.5, 25, 50, 100, 200, and 500 μg ml^−1^. At the same time, the cytotoxicity of solvents was examined. The control cell culture was supplemented with media including 2 % of serum only. The cell cultures were incubated for 48 h at 37 °C in the 5 % CO_2_ atmosphere. Cytotoxicity of the tested compound was estimated using the MTT method, described by Takenouchi and Munekata (1998). The MTT method is a quantitative colorimetric toxicity test, based on the transformation of yellow, soluble tetrazolium salts (3-[4,5-dimethylthiazol-2-yl]-2,5-diphenyltetrazolium bromide) to purple-blue insoluble formazane. This process occurs naturally in mitochondria of living cells. After 48 h incubation with compounds, cell cultures were supplemented with 10 μl of 5 mg ml^−1^ MTT solution per well, and further incubated for 4 h at 37 °C. Afterwards, 100 μl of water solution, including 50 % dimethylformamide and 20 % SDS, per well was added and after the all-night incubation the absorbance was measured by the 96-well plastic plate reader (Organon Teknika) at wavelengths of λ = 540 and 620 nm. The medium with DMSO at tested concentration range without the tested compound served as control––it was not toxic to vero cells line. The experiments were carried out in duplicates.
